# Supplementary material for: Angle-based wavefront sensing enabled by the near fields of flat optics
Source: Nat Commun. 2021 Oct 14;12:6002. doi: 10.1038/s41467-021-26169-z (PMC8516895; doi:10.1038/s41467-021-26169-z)
Supplement: Supplementary file 1 — Supplementary Info [file 41467_2021_26169_MOESM1_ESM.pdf]

Supplementary Information for

Angle-based wavefront sensing enabled by the near fields of flat optics

Soongyu Yi[1], Jin Xiang[1], Ming Zhou[1], Zhicheng Wu[1], Lan Yang[2], Zongfu Yu*[1]

1. Department of Electrical and Computer Engineering, University of Wisconsin, Madison
2. Department of Electrical and System Engineering, Washington University in St. Louis

*Email: zyu54@wisc.edu

Supplementary Note 1: Field calculation using Rayleigh-Sommerfeld diffraction theory

The field under the aperture can be calculated using Rayleigh-Sommerfeld diffraction theory

$$U(P_0) = -\frac{1}{2\pi}\iint_{\Sigma} U \frac{\partial G}{\partial n} ds \quad \text{(S1)}$$

where $\frac{\partial G}{\partial n} = cos(\vec{n}, \overrightarrow{r_{01}}) \left(jk - \frac{1}{r_{01}}\right) \frac{e^{(ikr_{01})}}{r_{01}}$. Here G is the Green function defined by the Helmholtz equation. $\boldsymbol{n}$ represents the normal direction of the integral area Σ, i.e. the aperture shown in Fig. S1. Here r_{01} is the distance between the point P_0 and the point P_1 on the area Σ, and k is the wavevector.

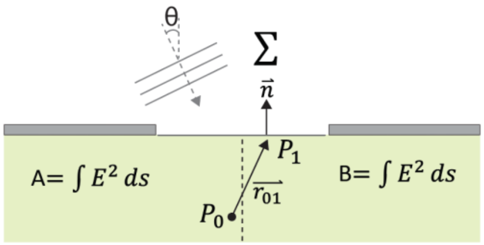

Fig. S1 Schematic of single slit aperture for calculating field energy using Rayleigh-Sommerfeld diffraction theory.

Supplementary Note 2: Energy distribution calculated by full-wave simulation

We calculate the energy ratio (A-B)/(A+B) between two symmetric regions A and B beneath an aperture (See Fig. S1) as a function of incident angle (θ in degree). The calculation is done for four different aperture sizes ranging from 0.5 μm to 2.0 μm and for five different wavelengths (450 nm, 500 nm, 550 nm, 600 nm, and 650 nm) using 2D full-wave simulation. For a fixed aperture, the wavelength dependence is weak, particularly for large apertures.

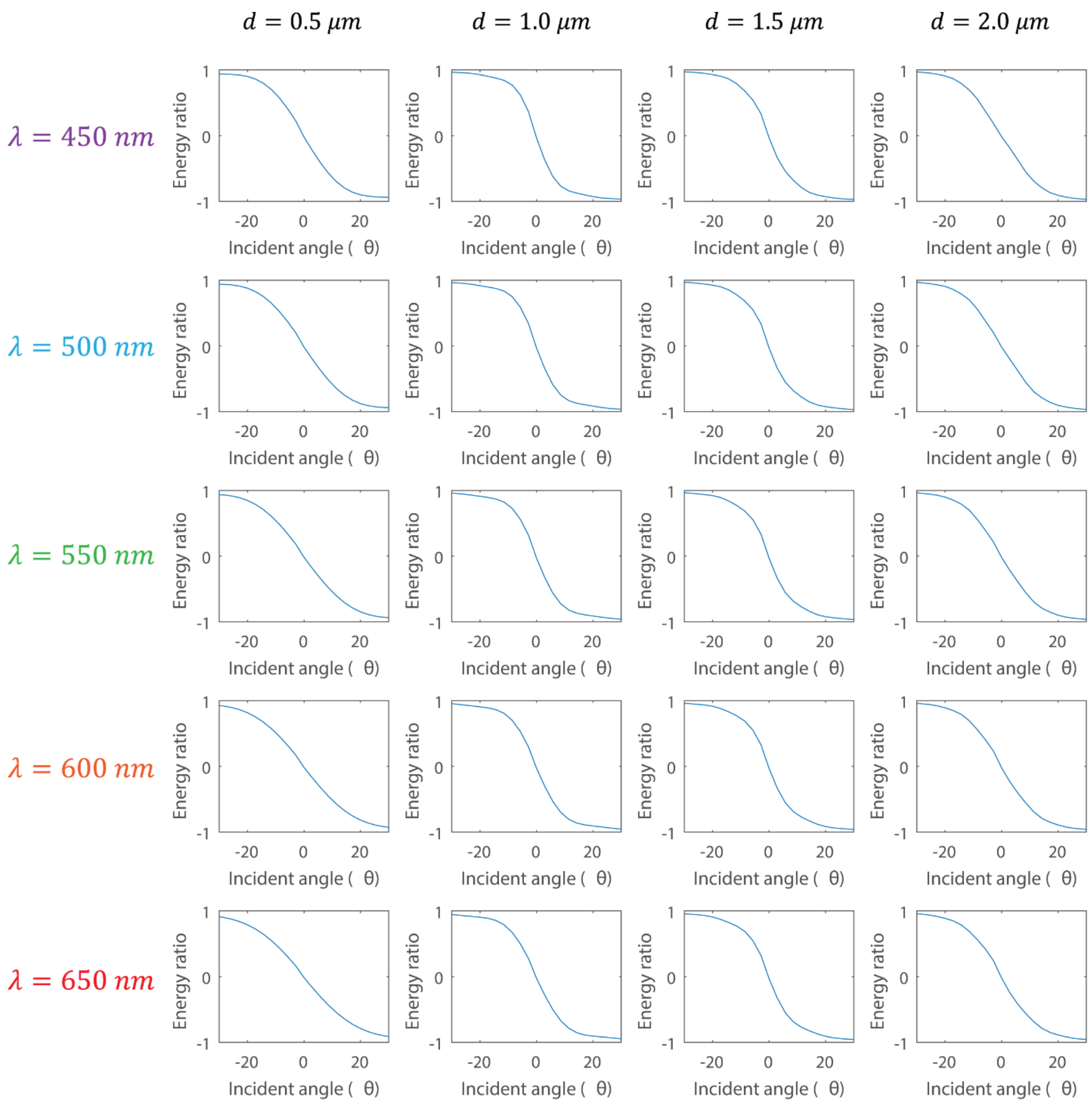

Fig. S2 Plot of energy ratio between two regions A and B depicted in Fig. S1 as a function of incident angle. Aperture size (d) and the wavelength of light (λ) are varied to calculate for different geometry.

The angular response of energy ratio exists even for a large aperture with a reduced angular sensitivity. The schematic of an actual device is shown in Fig. S3a. A CMOS image sensor with 5.2 μm pixel is used. We assumed 1μm thickness of SiO_2 passivation layer and 70 nm thickness of Si_3N_4 anti-reflective coating layer on top of Si layer which is a more realistic structure of CMOS image sensor. Thickness of the Al mask on top of the structure is 100 nm. Energy ratio of the 3D

structure was calculated using Tidy3D FDTD simulation tool [1]. As we see in Fig. S3b, energy ratio is incident angle dependent.

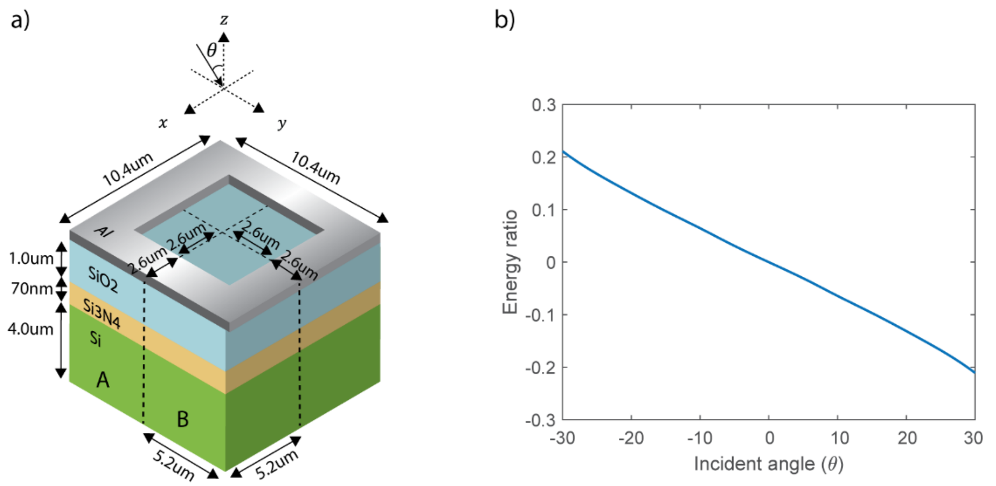

Fig. S3 a) 3D schematic with dimensions of the actual device. Thickness of the Al mask on top of the structure is 100 nm. b) Simulated energy ratio of the structure in a) using Tidy3D FDTD simulation tool.

Supplementary Note 3: Calibration of Wavefront Sensor

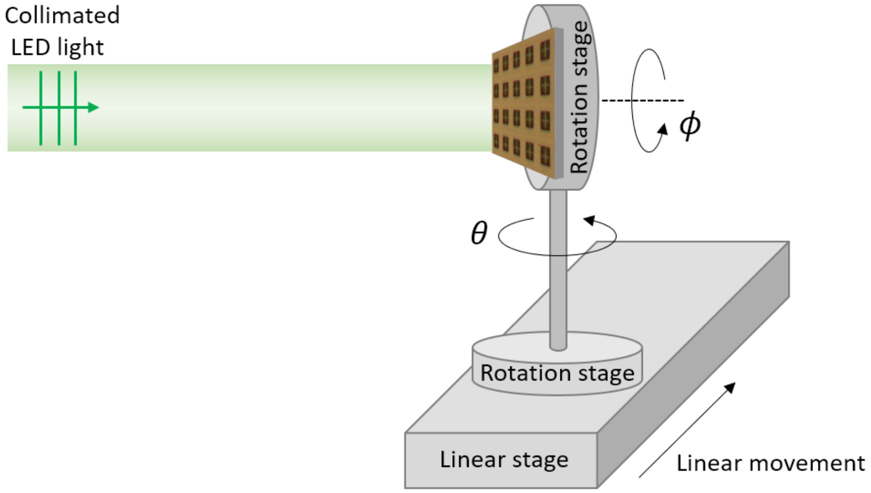

Fig. S4 Calibration setup of a wavefront sensor using a collimated LED light source and multiple stages to rotate the wavefront sensor in θ and ϕ direction.

The fabricated wavefront sensor was calibrated under a collimated LED light source. Two rotation stages and one linear stage were connected together as shown in Fig. S4 to rotate the sensor in θ and ϕ direction. The sensor was attached to the top rotation stage. The top rotation stage moves the camera in ϕ direction and the bottom rotation stage moves the camera in θ direction. A linear stage is used to compensate the off-axis movement of the wavefront sensor when it rotates in θ direction.

While the stage rotates, it changes the angle, θ and ϕ, between the collimated beam and the sensor. 30 raw image signals were recorded and the average was taken at each angle where $0^{\circ} \leq \theta \leq 30^{\circ}$ and $0^{\circ} \leq \phi \leq 360^{\circ}$ with a step of $\Delta\theta = \Delta\phi = 1^{\circ}$. This process is very efficient with a motorized stage and with all 62,500 angle sensors on the fabricated chip being calibrated at the same time.

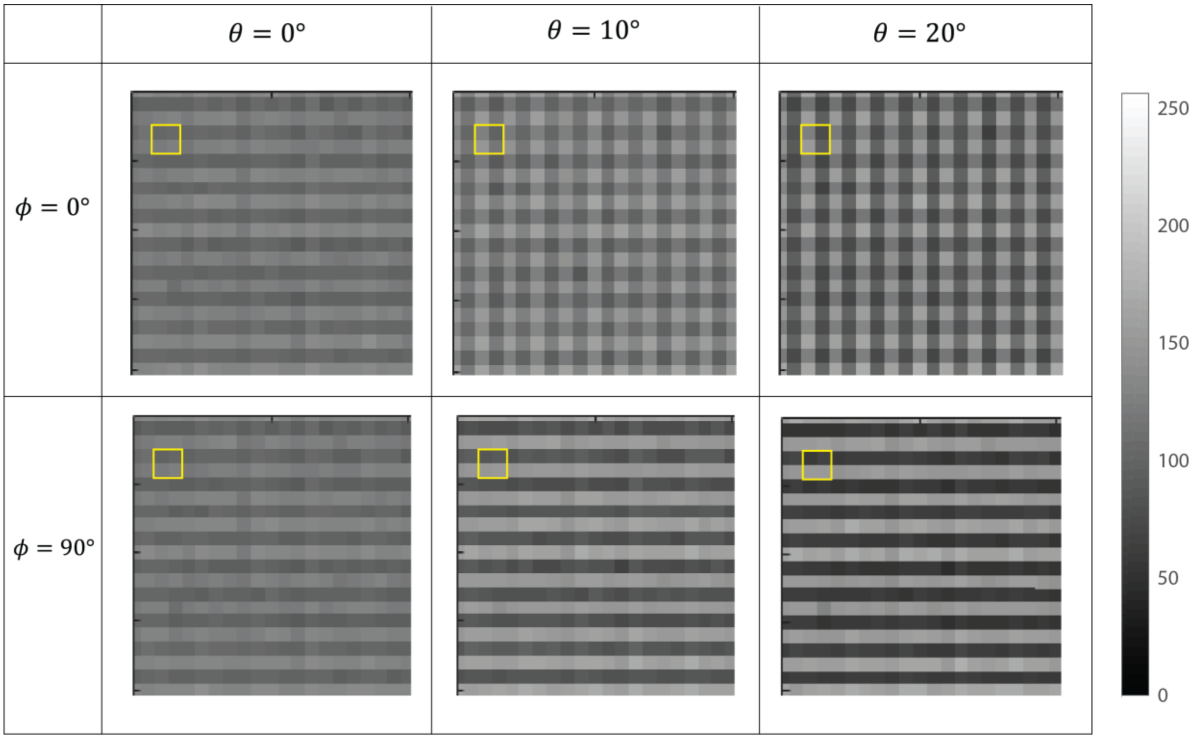

Fig. S5 Example of raw images captured by the wavefront sensor during the calibration process. Six images are shown as an example for different angles.

We present selected raw images captured during the calibration process to show the working principle of angle sensitive pixels. Each panel in Fig. S5 depicts a cropped region of the raw image captured at different incident angles. The camera uses an 8-bit ADC (analogue-to-digital converter) meaning that the minimum intensity is 0 (black pixel) and the maximum intensity is 255 (white pixel). Here, we are able to observe the angle dependency between neighboring pixels. The yellow square represents a super-pixel that consists of 2x2 pixels.

As θ increases while $\phi = 90°$, image contrast will increase in vertical direction while the contrast does not change in horizontal direction. This is because the projected LED location changes in vertical direction. For the actual measurement, a slight contrast change exists in horizontal direction due to the misalignment between the fabricated Al layer and the pixel layer. However, this misalignment is embedded in the raw image thus it has no effect to the sensor performance. The same can be observed while $\phi = 0°$ and θ increases. In this case, the image contrast exists in lateral while the contrast is minimum in vertical direction. Once the calibration is done for the entire range of θ and ϕ, acquired raw images will be used as a look up table for individual angle sensing pixels to determine the incident angle of light.

Supplementary Note 4: Spherical wavefront measurement

This section discusses the evaluation of the wavefront sensor using a spherical wave. A lens with a 25 mm focal length was used to generate a divering beam. A wavefront of a diverging beam was measured at 3 different locations A, B, and C. As the wavefront sensor is placed further away from the focal point, measured radius of curvature (ROC) of the spherical wavefront will decrease. Measured wavefronts at position A, B and C are shown in Fig. S6. It is clear that the wavefront measured further away from the focal point has less curvature. ROC was calculated for all three wavefronts and they are compared with theoretical ROC in table S1. The measured ROC and theoretical ROC are in good agreement.

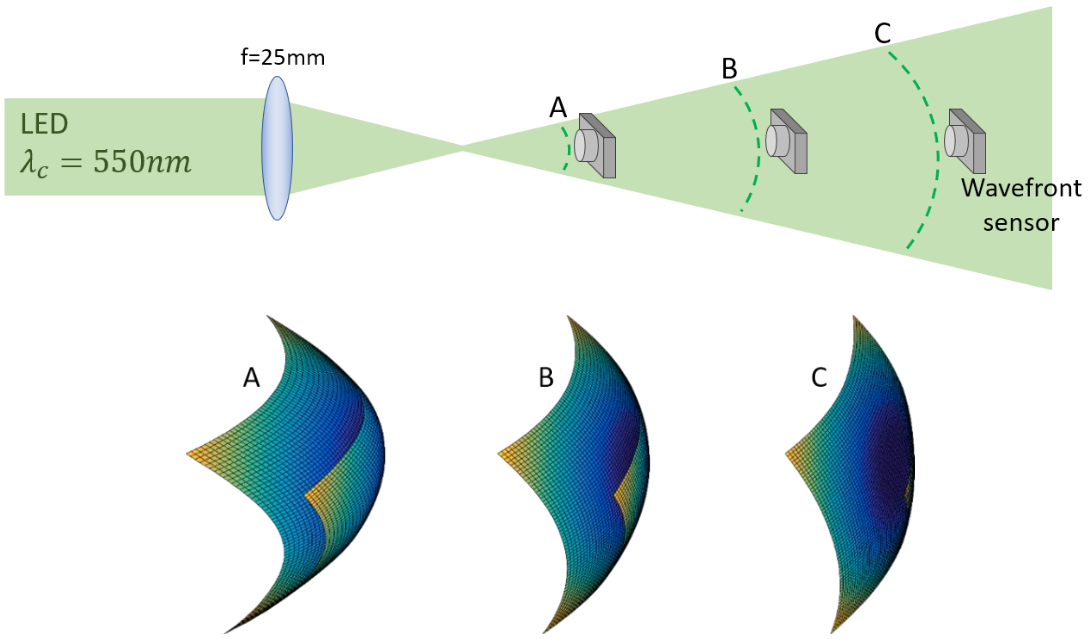

Fig. S6 Schematic of spherical wavefront measurement using our wavefront sensor. Measurement was done at three different locations. The calculated wavefronts at position A, B, and C are shown.

Position	Theoretical ROC	Measured ROC
A	3 mm	3.14 mm
B	5 mm	5.08 mm
C	7 mm	7.08 mm

Table S1. Comparison between theoretical ROC and measured ROC at three different positions. Both are in good agreement.

Supplementary Note 5: Conversion of angle measurement to wavefront

Measured wavefront can be used to calculate the surface height of the PMMA. The phase delay $\varphi(x, y)$ at each unit pixel is related to the height information $h(x, y)$ at each unit cell as [2]

$$\varphi(x, y) = h(x, y)\frac{2\pi}{\lambda}\Delta n \qquad \text{(S2)}$$

where λ is the wavelength of incident light and Δn is the index difference between the sample and the surrounding medium. We use zonal estimation [3] to reconstruct the wavefront from $\varphi(x, y)$ calculated at each unit pixel.

Supplementary Note 6: Reflection Mode

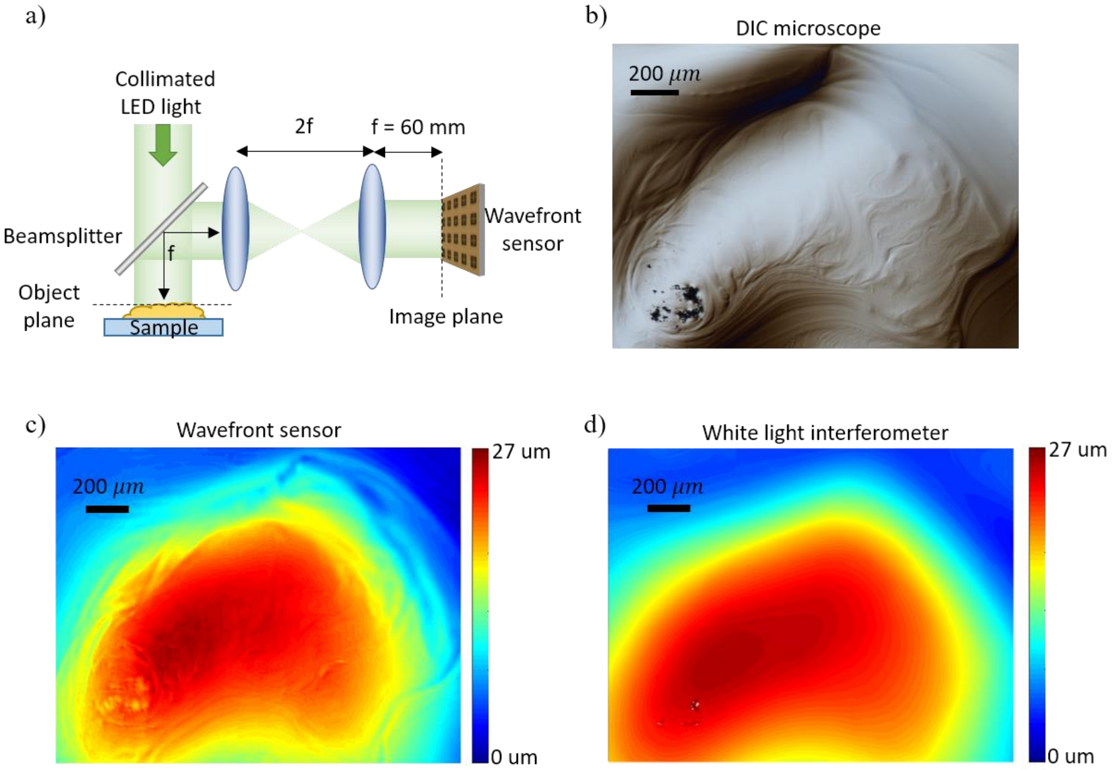

Fig. S7 a) Surface profile measurement setup for reflection mode. b) Optical image of a coagulated PMMA polymer obtained from a differential interference contrast microscope. c) Surface profile measured with the optical setup in a) with our wavefront sensor. d) Measured result using a commercial white light interferometer.

Surface profile of a non-transparent sample can be measured under a reflection mode setup as shown in Fig. S7a. This setup simply adds a beam splitter to an existing transmission mode setup.

For sample preparation, a PMMA polymer sample was coated with 10 nm Al to make its surface reflective.

Figure S7b is a microscope image taken under a differential interference contrast (DIC) mode. While the image does not deliver any quantitative information about the surface profile, it does provide a qualitative information of the surface profile gradient.

Figure S7c shows a wavefront sensor measured surface profile using the reflection mode setup. Not only does it show the surface profile but also delivers the actual height information which DIC microscope does not. The height difference between local maximum and local minimum within the captured frame is around 27 μm. Figure S7d shows the same region of the sample measured with a white light interferometer (WLI). WLI is not able to capture the surface roughness details that are clearly visible in DIC mode and wavefront sensor measurement results.

Supplementary Note 7: Minimum and maximum detectable angle of a wavefront tilt

The minimum detectable angle $\delta\theta$ of the wavefront sensor can be expressed as

$$\delta\theta = \Delta R \cdot \frac{d\theta}{dR} \quad (S3)$$

where R is the pixel intensity ratio between two neighboring pixels. If we assume R has a linear response up to $\theta = D$ as shown in Fig. S8a, $d\theta/dR$ can be expressed as

$$\frac{d\theta}{dR} = \frac{D}{R_{max} - 1} \quad (S4)$$

where D is the maximum angle that wavefront sensor has a linear response and R_{max} is the maximum pixel intensity ratio at D degree.

Here, we show how minimum detectable angle around normal incidence can be calculated. Since $R = P_1/P_2$ where P_1 and P_2 are pixel intensity of two neighboring pixels, ΔR can be expressed as:

$$\Delta R = \left|\frac{dR}{dP_1}\right| \Delta P_1 + \left|\frac{dR}{dP_2}\right| \Delta P_2 = \left|\frac{1}{P_2}\right| \Delta P_1 + \left|\frac{P_1}{P_2^2}\right| \Delta P_2 \quad (S5)$$

Since $P_1 \approx P_2$ when light is normal incident, Eq. S5 can be written as

$$\Delta R = \left|\frac{1}{P_1}\right| \Delta P_1 + \left|\frac{1}{P_2}\right| \Delta P_2 = \frac{2}{SNR} \quad (S6)$$

Thus, substituting Eq. S4 and S6 into Eq. S3, $\delta\theta$ can be expressed as

$$\delta\theta = \frac{2}{SNR} \cdot \frac{D}{R_{max} - 1} \tag{S7}$$

If we assume SNR $= 45$ dB and use $R_{max} = 1.1$ and $D = 5°$ which is based on our experimental results in Fig. S8b, $\delta\theta$ can be calculated as

$$\delta\theta = \frac{2}{10^{4.5}} \cdot \frac{5°}{1.1 - 1} \approx 0.0032° \tag{S8}$$

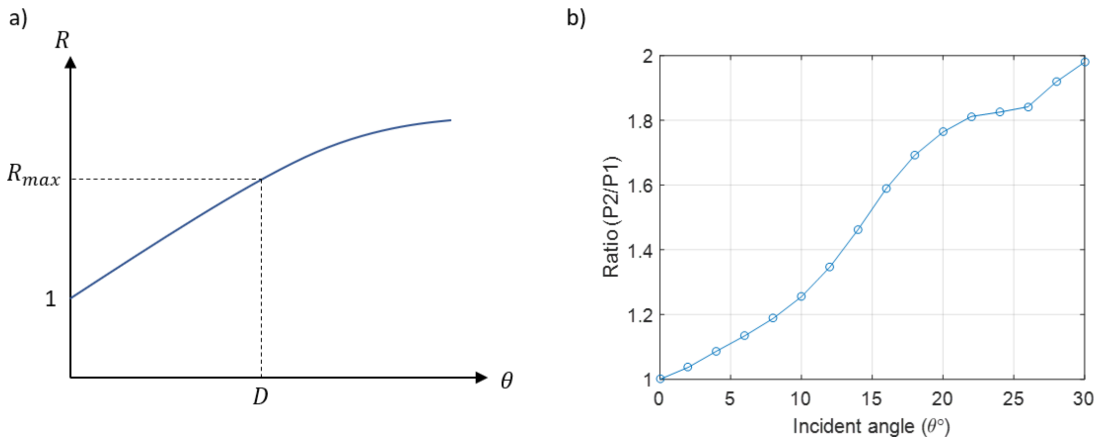

Fig. S8 a) Pixel intensity ratio (R) of two neighboring pixels as a function of incident angle (θ). b) Measured pixel intensity ratio of two neighboring pixels. The neighboring pixels were randomly selected within the fabricated wavefront sensor.

Next, we discuss the maximum detectable angle, θ_{max}. For a lens based system such as Shack-Hartmann wavefront sensor, θ_{max} depends on the focal length and the diameter of a lens. Figure S9 illustrates θ_{max} for a lens based system. θ_{max} is equivalent to the maximum displacement of the focal spot with respect to normal incident case. As shown in Fig. S9b, maximum focal spot shift with respect to normal incident case (Fig. S9a) is around $d/2$. Once the focal spot shift exceeds $d/2$, there will be an uncertainty for mapping out the correct lens that has generated the focal spot of interest. For a typical Shack-Hartmann wavefront sensor (Thorlabs WFS20-5C), $\theta_{max} \approx 1°$ with a spatial resolution of 150 μm which is equivalent to lenslet pitch. θ_{max} can be larger than 1° at the cost of spatial resolution since it will require larger d.

Unlike a lens-based system, our wavefront sensor is based on flat optics that has much more flexibility of θ_{max}. As shown in Fig. S8b, θ_{max} of our wavefront sensor can reach up to 30° with a spatial resolution of only 2 pixels (10.4μm). θ_{max} is 30 times higher and spatial resolution is almost 15 times higher compared to a commercial Shack-Hartmann wavefront sensor.

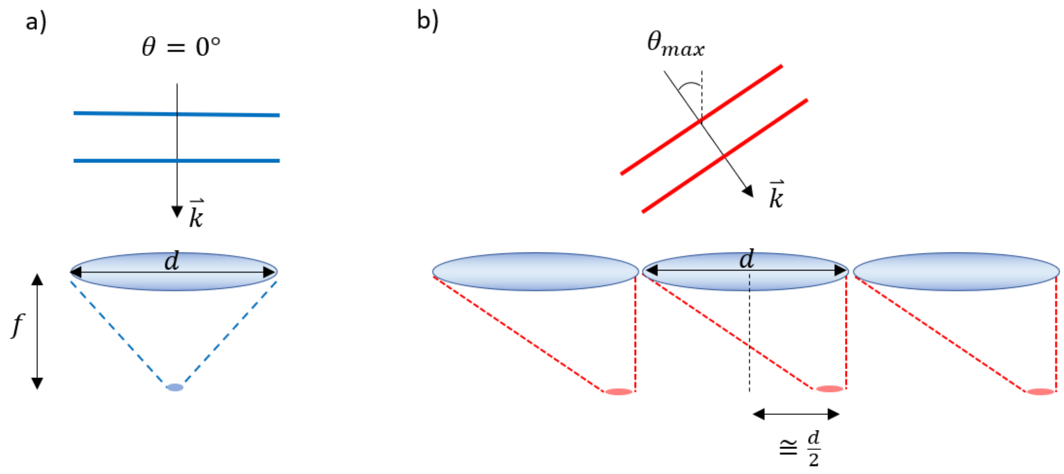

Fig. S9 a) When a lens is used to focus a normal incident light, location of the focal spot will not shift. It will be below the center of a lens. b) For an oblique incident light, the focal spot will shift. The amount of shift depends on the incident angle. Maximum detectable angle (θ_{max}) for a lens based system is limited by the lens diameter.

Supplementary Note 8: Analysis of performance gain compared to WLI

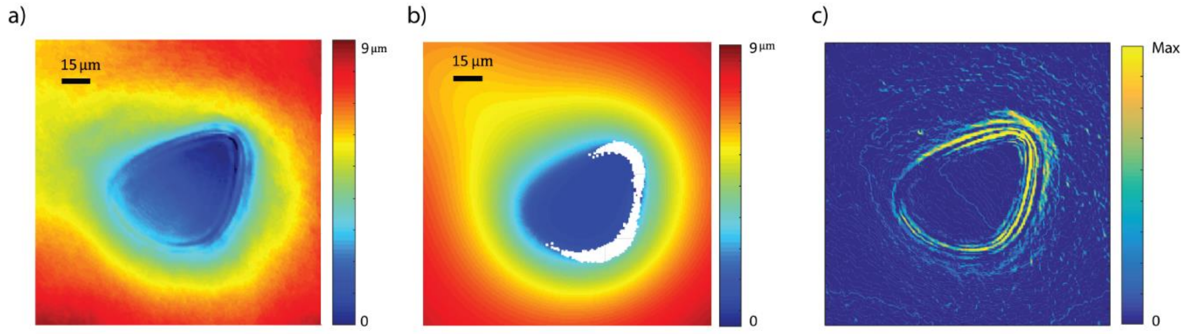

Fig. S10 a) Surface profile of a PMMA sample measured with our wavefront detector. b) Measurement result using WLI. Side wall regions cannot be measured. c) Magnitude of the gradient calculated from the result in Fig. S10a. As expected, gradient magnitude of sidewall shows large value compared to flat region.

The region where WLI fails to measure can be understood by looking at the surface slope. Fig. S10a shows the surface profile of a PMMA sample measrued with our wavefront detector and Fig. S10b shows the same region measured with WLI (Zygo NewView 9000). The measruement region WLI tend to fail is the side wall region where the surface slope is high and thus the interference pattern is not captured by the image sensor integrated with WLI. Fig. S10c shows the magnitude of the gradient calculated from the measurement result in Fig. S10a. Blue region indicates flat region and yellow region indicates the region with steep slope of the sureface profile. By

comparing Fig. S10b and S10c, we can see that the region WLI fails to measure is the region where the surface profile gradient is high relative to the flat surface.

Supplementary Note 9: Image comparison with WLI and DIC microscope

Here, we compare the surface profile taken with three different tools. Figure S11a is taken with WLI. While it measures the overall height accurately, it will not reveal the detailed surface roughness. Using our wavefront sensor, the details can be revealed as in Fig. S11b which is consistent with the image taken using a differential interference contrast (DIC) microscope as in Fig. S11c. Note that DIC only delivers a qualitative image meaning that the image contrast does not accurately represent the surface profile. It provides an artificial 3D-like surface profile. Figure S11d and S11e shows the angular component of Fig. S11b in x and y direction, respectively. These two images convey similar information as that of a DIC microscope image since they only show an angular gradient in one specific direction which can correspond to the polarization angle of DIC microscope.

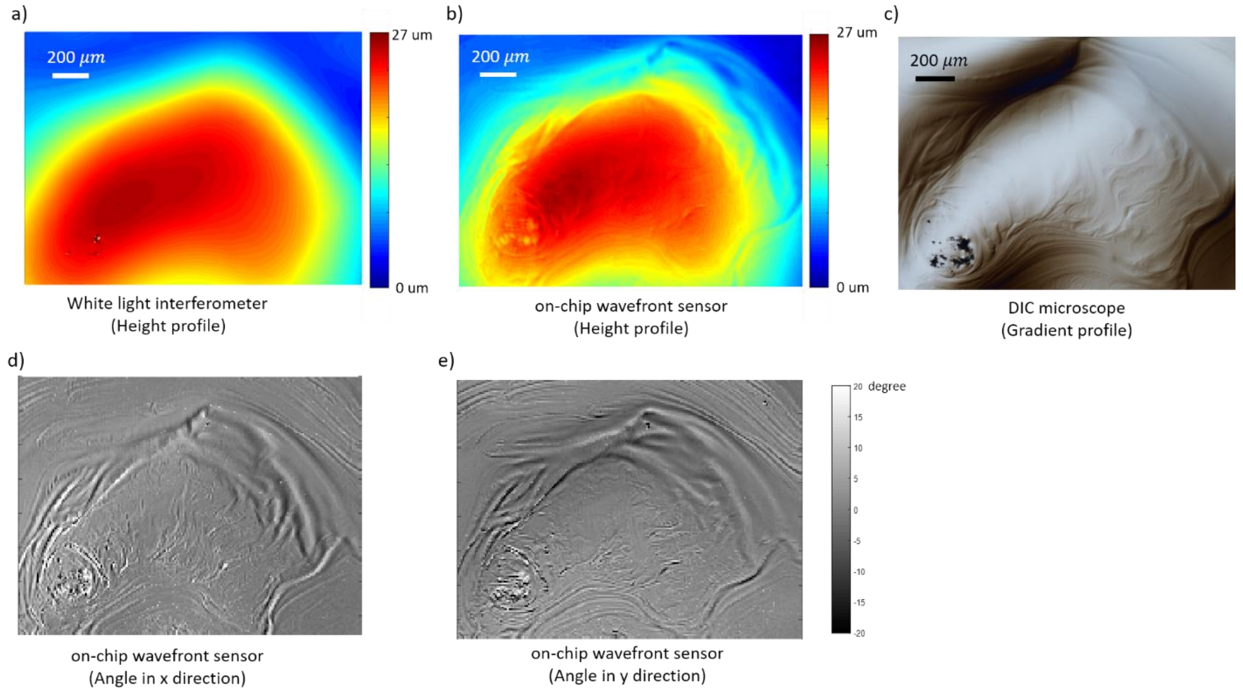

Fig. S11 Surface profile measurement using a) white light interferometer and b) our wavefront sensor. c) Gradient profile measured with DIC microscope d) incident angle projected on x-axis direction. e) Incident angle projected on y-axis direction.

Supplementary Note 10: Length scale comparison with conventional Hartmann sensor

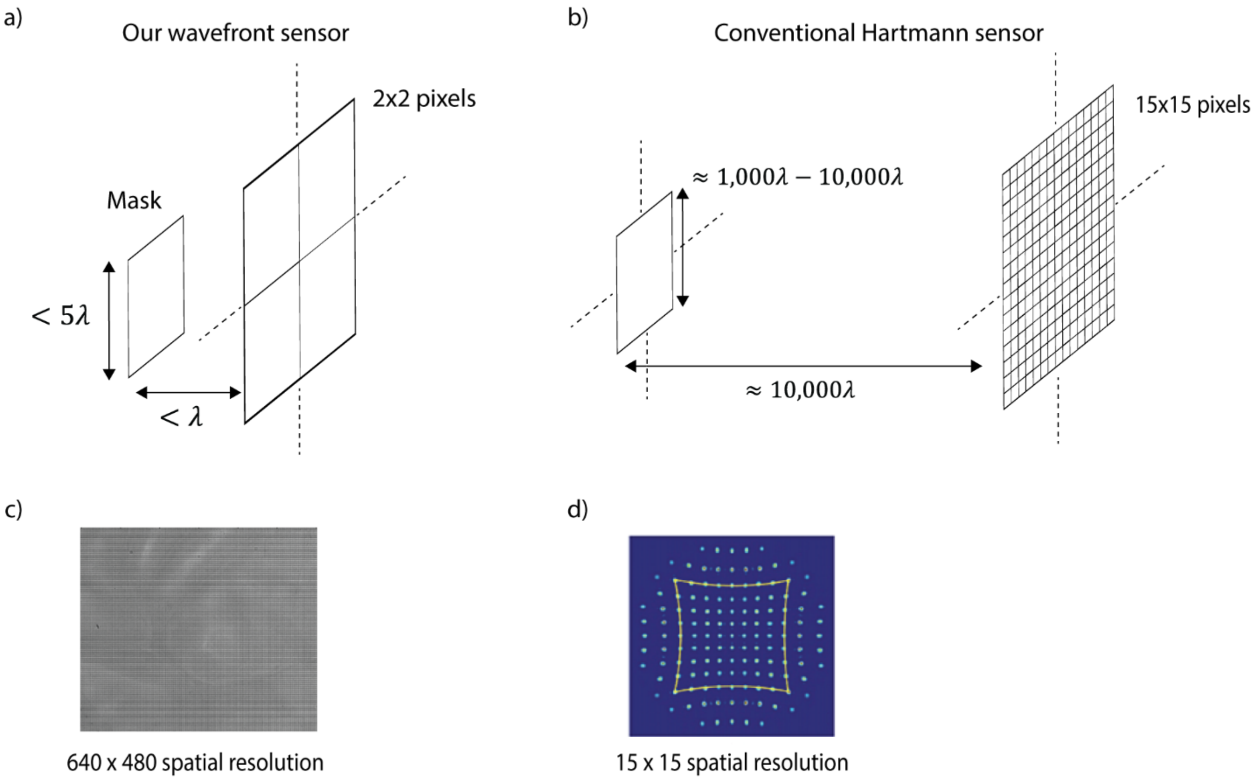

Fig. S12. a) Schematic of our wavefront sensor with extremely small mask and short distance between mask and sensor layer. b) Schematic of conventional Hartmann sensor with extremely large aperture and mask to sensor distance. c) Example of raw measurement data captured with our wavefront sensor. d). Raw measurement data of conventional Hartmann sensor cited from [4] only showing 15x15 spatial resolution.

People have used apertures to measure wavefronts in EUV [5] and X-ray [4]. This is due to the lack of lens in these wavelength ranges. However, being micro-lens in the visible range or apertures in the EUV/X-ray range, traditional Hartmann sensors follow the same operating principle, and it is different with our sensor in one important aspect: length scale.

The difference in length scale leads to different regimes for the wave physics and the resulting performance metric is also drastically different. The aperture in EUV/X-ray, are 1000 – 10,000 times of wavelength. The distance between the aperture and the sensor plane is even larger (Fig. S12b). In contrast, all length scale in our system is all around wavelength scale (Fig. S12a). This difference dictates that the two systems explore different physics: the former primarily relies on ray optics with far-field diffraction correction. The latter, i.e. our sensor, needs full wave electrodynamics and exploit near-field energy distribution. Because we explore a quite different length scale and primary physical mechanism, the new system can realize a performance that is orders of magnitude better in both spatial resolution and angular dynamic range. Note the spatial resolution difference between our sensor (Fig. S12c) and conventional Hartmann sensor (Fig. S12d).

Consequently, this performance improvement enables the angle-based approach to be used for quantitively phase imaging, a new area of angle-based sensor. This further enables a new capability for real-time video recording of microscopic phase front. Such new capability is highly desired for bio imaging and material characterization.

Supplementary References

[1] Tidy3D, https://simulation.cloud

[2] J. W. Goodman, Introduction to Fourier Optics (Roberts and Company, 2005).

[3] Southwell, W. H. Wave-front estimation from wave-front slope measurements. *JOSA* **70**, 998–1006 (1980).

[4] Cocco, D., Idir, M., Morton, D., Raimondi, L. & Zangrando, M. Advances in X-ray optics: From metrology characterization to wavefront sensing-based optimization of active optics. *Nucl. Instrum. Methods Phys. Res. Sect. Accel. Spectrometers Detect. Assoc. Equip.* **907**, 105–115 (2018).

[5] Mercère, P., Zeitoun, P., Idir, M., Le Pape, S., Douillet, D., Levecq, X., Dovillaire, G., Bucourt, S., Goldberg, K.A., Naulleau, P.P., and Rekawa, S., Hartmann wave-front measurement at 13.4 nm with λ_{EUV}/120 accuracy, Opt. Lett. 28, 1534-1536 (2003).
