# Supplementary material for: Angle-based wavefront sensing enabled by the near fields of flat optics
Source: Nat Commun. 2021 Oct 14;12:6002. doi: 10.1038/s41467-021-26169-z (PMC8516895; doi:10.1038/s41467-021-26169-z)
Supplement: Supplementary file 3 — Description of Additional Supplementary Files [file 41467_2021_26169_MOESM3_ESM.docx]

**Description of Additional Supplementary Files**

File name: Supplementary Movie 1

Description: **Surface topography in video frame rate.** Temporal dynamics of a coagulating PMMA polymer is recorded at 30 frames per second.
